# Determinants of Prolonged Length of Hospital Stay of Patients with Atrial Fibrillation

**DOI:** 10.3390/jcm10163715

**Published:** 2021-08-20

**Authors:** Ancuța Elena Vîjan, Ioana Cristina Daha, Caterina Delcea, Gheorghe-Andrei Dan

**Affiliations:** 1 Internal Medicine and Cardiology Department, Carol Davila University of Medicine and Pharmacy, 020021 Bucharest, Romania; ancuta.vijan@drd.umfcd.ro (A.E.V.); ioana.daha@umfcd.ro (I.C.D.); andrei.dan@gadan.ro (G.-A.D.); 2 Cardiology Department, Colentina Clinical Hospital, 020125 Bucharest, Romania

**Keywords:** atrial fibrillation, extended hospitalization, HAS-BLED, heart failure, length of hospital stay

## Abstract

Background and Aim: The increasing prevalence and high hospitalization rates make atrial fibrillation (AF) a significant healthcare strain. However, there are limited data regarding the length of hospital stay (LOS) of AF patients. Our purpose was to determine the main drivers of extended LOS of AF patients. Methods: All AF patients, hospitalized consecutively in a tertiary cardiology center, from January 2018 to February 2020 were included in this retrospective cohort study. Readmissions were excluded. Prolonged LOS was defined as more than seven days (the upper limit of the third quartile). Results: Our study included 949 AF patients, 52.9% females. The mean age was 72.5 ± 10.3 years. The median LOS was 4 days. A total of 28.7% had an extended LOS. Further, 82.9% patients had heart failure (HF). In multivariable analysis, the independent predictors of extended LOS were: acute coronary syndromes (ACS) (HR 4.60, 95% CI 1.66–12.69), infections (HR 2.61, 95% CI 1.44–3.23), NT-proBNP > 1986 ng/mL (HR 1.96, 95% CI 1.37–2.82), acute decompensated HF (ADHF) (HR 1.76, 95% CI 1.23–2.51), HF with reduced ejection fraction (HFrEF) (HR 1.69, 95% CI 1.15–2.47) and the HAS-BLED score (HR 1.42, 95% CI 1.14–1.78). Conclusion: ACS, ADHF, HFrEF, increased NT-proBNP levels, infections and elevated HAS-BLED were independent predictors of extended LOS, while specific clinical or therapeutical AF characteristics were not.

## 1. Introduction

Atrial fibrillation (AF) emerged as the leading cause for rhythm disorder hospitalizations [1]. With an increased prevalence over the past years, mainly due to the ageing population, as well as better survival after the first cardiovascular event and improved diagnostic tools, the burden on the healthcare system is on the rise [2].

The worldwide AF prevalence is 2% to 4%, and it is estimated to affect 46.3 million people globally [3]. In the European population, the lifetime risk of AF is evaluated to one in five middle aged adults without cardiovascular risk factors and to on in three individuals with multimorbidity [2,4].

While the AF associated mortality decreased over the last decade, the cost of hospitalization had an exponential rise, and the length of hospital stay (LOS) is still prolonged [5,6]. The annual AF hospital admission rate varies between 10 and 40% [7], with 30% of patients having at least one hospitalization per year, and 10% two or more [8]. The main causes of hospitalization and AF mortality include stroke, heart failure (HF), myocardial infarction and sudden cardiac death [9]. The risk of death among patients with AF increases 1.5, respectively 1.9 times in men and women aged over 55 years, after adjusting for other cardiovascular comorbidities [10].

The AF associated high burden of complications leads to increased and extended hospitalization rates. The risk of stroke is five times greater in nonvalvular AF patients [11]. Irrespective of which developed first, the interrelation of heart failure and AF is linked to higher risk of mortality and lower quality of life [12,13]. The association of the two pathologies and their impact on mortality depends on the timing of AF onset [14]. The risk of mortality is higher in patients with preexisting HF that develop AF [15], while in patients with preceding AF new onset HF modulates the mortality risk in a time-dependent manner [16]. In anticoagulated AF patients, concomitant HF is the leading cause of death [17].

Hospitalization in cardiac patients is influenced by several factors, including medical complexity, patient and hospital characteristics [18]. The wide variation of LOS in cardiovascular disease significantly alters healthcare outcomes [19]. LOS is a well-established parameter used to evaluate severity of illness, hospital performance, resource utilization and healthcare cost [20]. Previous studies showed that extended LOS is a marker of poor prognosis and risk of early rehospitalization in HF patients [21,22]. For AF patients, however, scarce data are available regarding prolonged hospitalization. We, therefore, aim to identify the determinants of extended LOS of AF patients, as an initial step in improving hospitalization outcomes.

## 2. Materials and Methods

### 2.1. Study Design and Population

This is an observational, retrospective cohort study performed in a tertiary cardiology center. The study protocol was approved by the hospital’s ethical board, in accordance with the Declaration of Helsinki.

We included all patients with AF, aged 18 years and older hospitalized consecutively to the Cardiology Department, from January 2018 to February 2020. We excluded all readmissions of the same patient. Demographic data, diagnoses and laboratory tests were extracted from the patients’ electronic medical records.

### 2.2. Definitions

According to the type of AF, patients were classified in three groups: paroxysmal, persistent and permanent. As the term of “long-term persistent AF” was seldom used for the diagnosis in our cohort, it was not included into definitions.

HF patients were classified, using current guidelines [23], according to the ejection fraction (EF) as follows: preserved EF (HFpEF), mid-range EF (HFmEF) and reduced EF (HFrEF).

The comorbidity burden and frailty was assessed according the Charlson comorbidity index [24].

The method of choice for cardioversion was electrical for all patients undergoing the procedure.

Prolonged LOS was defined as more than seven days (the upper limit of the third quartile), coinciding to methodology as well as cut-off value previously reported in similar studies [25,26].

### 2.3. Laboratory Measurements

Venous samples were collected on the day of the admission. Complete blood cell counts were analyzed with Abbott Celldyn 3700 (Abbott Laboratories Santa Clara, CA, USA) while biochemistry and immunology samples were evaluated with Hitachi Modular analyzer (Hitachi, Ltd., Tokyo, Japan). NT-proBNP was determined with Roche Diagnostics Elecsys^®^ assay (Roche, Indianapolis, IN, USA).

General Electric’s Vivid S6, GE Healthcare Wauwatosa, WI, USA and Philips Epiq 7, Koninklijke Philips N.V. Amsterdam, The Netherlands, were used to perform transthoracic echocardiography by experienced staff physicians.

### 2.4. Statistical Analysis

IBM SPSS Statistics 23 (IBM Corp. Armonk, NY, USA), Epi Info 7 (CDC, USA) and MedCalc Statistical Software version 19.0.7 (MedCalc Software bvba, Ostend, Belgium) were used for statistical analysis.

Non-parametric data were expressed as median with interquartile range and normally distributed data as mean with standard deviation. Categorical data were expressed as absolute numbers and percentages.

The Student’s *t* test, ANOVA and Mann-Whitney-Wilcoxon tests were used to compare independent continuous variables. Yates’ corrected Chi-square test was used for categorical variables with dichotomous values, and Chi-square test for trend for categorical variables with three or more values.

ROC curve analysis was performed to test the correlations between numerical and categorical variables. Youden index associated criterion was used to determine optimal cut-off values for the continuous variables associated with the outcome.

The independent associations between determinant variables and the outcome were performed using multiple logistical regression, forward and backward conditional methods to ensure accuracy. All parameters significantly associated with the outcome in univariate analysis were included in the multivariable analysis. NT-proBNP was included in the regression as a dichotomous variable, using the cut-off value obtained in ROC analysis.

We considered a *p*-value < 0.05 to be statistically significant.

## 3. Results

### 3.1. Study Population

**Figure 1:**
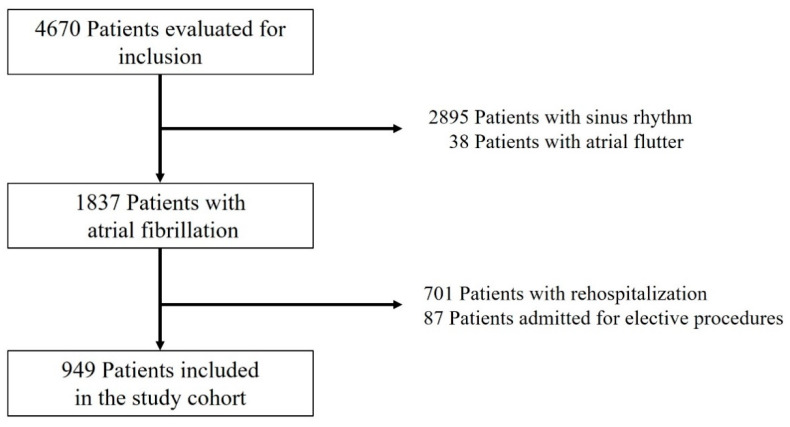
PRISMA consort flow diagram of the study.

Our study included 949 AF patients (Figure 1, Table 1). A total of 52.9% were females. The mean age was 72.5 ± 10.4 years. The median LOS was 4 days. A total of 28.7% had an extended LOS. HF was present in more than 80% patients, of which more than half had HFpEF. Mean EF was 45.8 ± 13.8%.

Common comorbidities included ischemic heart disease (IHD) (32.4%), arterial hypertension (80.6%), diabetes mellitus (31.3%), dyslipidemia (68.0%), obesity (31.1%) and chronic kidney disease (36.0%). A total of 14.5% of patients had a history of stroke or transient ischemic attack (TIA). The median CHA2DS2-VASc score was 4 and HAS-BLED 1. Of the three types of AF, permanent AF was prevalent (44.8%) (Table 1).

Patients with permanent AF were older and had longer hospitalizations. They had a significantly higher prevalence of HF, larger left atrial diameters and lower EF (Table S1). Patients admitted with ACS were more likely to have paroxysmal AF rather than permanent AF (*p* = 0.02). Hypertension, obesity and diabetes mellitus had similar prevalence irrespective of AF type (Table S1). No association between history of stroke/TIA and type of AF was observed (*p* = 0.43). Patients with paroxysmal and persistent AF receiving anticoagulant therapy were prescribed NOACs more frequently than VKA, compared to permanent AF (*p* < 0.001) (Table S1).

### 3.2. Length of Hospital Stay

Patients with extended LOS were older, with more comorbidities, predominantly associating HF and increased NYHA class, and more likely to have permanent AF. They also had a significantly higher prevalence of ischemic heart disease, including prior myocardial infarction, history of stroke, diabetes mellitus, chronic kidney disease and dementia (Table 1).

**Figure 2:**
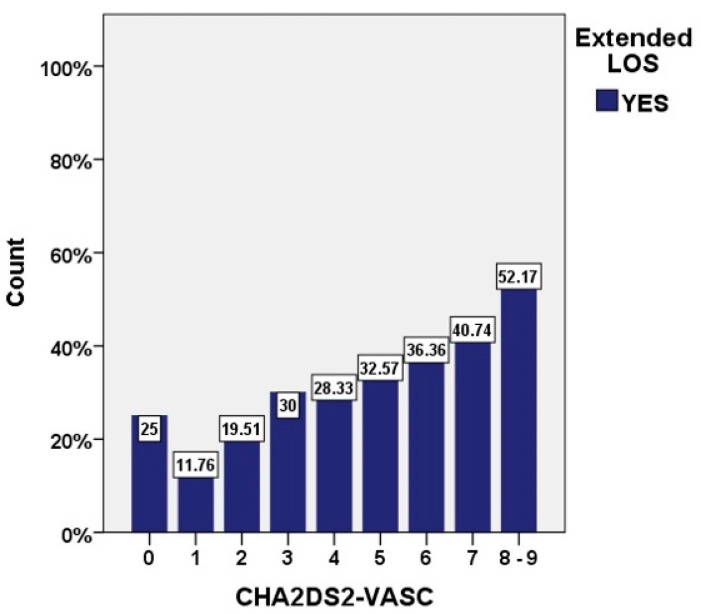
Incidence of prolonged LOS stratified by the CHA_2_DS_2_-VASc score.

**Figure 3:**
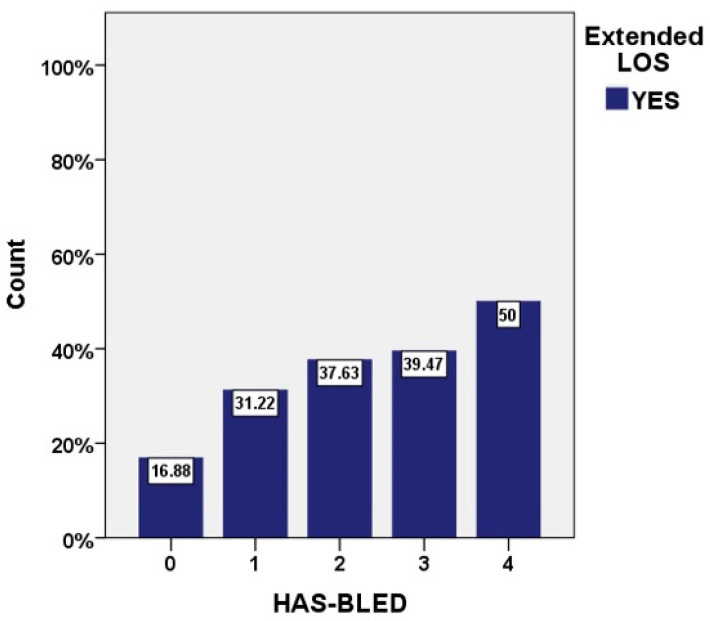
Incidence of prolonged LOS stratified by the HAS-BLED score.

Incidence of prolonged LOS was proportional to the CHA_2_DS_2_-VASc score, ranging from 11.8% for patients with 1 point to 52.2% for patients with 8 or 9 points (*p* for trend < 0.001) (Figure 2), as well as to the HAS-BLED score, ranging from 16.9% for patients with 1 point to 50% for patients with 4 points (*p* for trend < 0.001) (Figure 3). Most conditions included in the CHA_2_DS_2_-VASc score and almost half of those composing the HAS-BLED score represented risk factors for prolonged hospitalization (Table S2).

Vitamin K antagonists were more prevalent in patients with extended LOS, compared to NOACs. Antiplatelet therapy had similar use in the two groups (Table 1). Emergency cardioversion did not influence LOS (Table 1).

In univariate analysis, extended hospitalization was associated with the presence of HF and markers of HF severity (dyspnea at rest, ADHF, NT-proBNP with a cut-off value > 1986 pg/mL and lower EF—cut-off value < 44%) and IHD (ACS, *p* < 0.001; prior myocardial infarction, *p* < 0.001; chest pain on admission, *p* = 0.04). Regarding the clinical course of AF, emergency cardioversion did not influence LOS (Table 1 and Table 2). Patients with AF at admission had longer hospital stay (*p* = 0.04), while patients presenting for palpitations had a shorter LOS (*p* = 0.02), as did patients with hypertensive emergencies (*p* = 0.005). Non-cardiac conditions correlated with extended LOS were infections (*p* < 0.001), dementia (*p* < 0.001), history of stroke or TIA (*p* = 0.01) and decreased renal function (*p* = 0.01) (Table 2 and Table 3).

In multivariable regression analysis (Table 4), after adjusting for age and sex, we identified five independent predictors of extended LOS. ACS (HR 4.60, *p* = 0.003) was the strongest predictor of prolonged hospitalization followed by coexisting infections, (HR 2.61, *p* < 0.001), NT-proBNP > 1986 pg/mL (HR 1.96, *p* < 0.001), ADHF (HR 1.76, *p* = 0.002), presence of HFrEF (HR 1.69, *p* = 0.007) and increased HAS BLED score (HR 1.42, *p* < 0.001).

## 4. Discussion

One of the few studies to evaluate extended LOS in AF patients, our research identified several determinants of prolonged hospitalization in a relatively large cohort of AF patients. A detailed characterization of hospitalization determinants of a highly prevalent cardiac disease represents an initial step in improving patient outcomes as well as healthcare burden and associated costs.

Mean LOS was four days in our sample, comparable with previously reported results. In an analysis of AF patients hospitalized in the US over a period of eleven years, the mean LOS was three days, with unchanged yearly data over the duration of the study [27].

Our study identified several determinants of prolonged LOS in AF patients, related to stroke and bleeding risk, severity of AF burden and cardiovascular altered substrate.

### 4.1. Risk Scores in Atrial Fibrillation

The correlation between the CHADS2 score and LOS was documented in AF patients [28], and the increase of LOS was shown to be directly proportional to both CHADS2 and CHA_2_DS_2_-VASc scores [29]. While both CHA_2_DS_2_-VASc and HAS-BLED scores have been correlated to mortality in AF patients [30,31], the latter was not previously studied in relation to LOS. Our results not only confirm the association between duration of hospitalization and the CHA2DS2-VASc score, but also establish the independent predictive value of the HAS-BLED score for extended LOS in AF patients. In our analysis, 5.45% patients had a high bleeding score, mostly with non-modifiable and partially modifiable risk factors (age, previous stroke, renal impairment, uncontrolled hypertension), and, as previously shown, timely management of bleeding risk is crucial in AF patients, being also an important driver of early readmissions [32].

Age was poorly correlated with extended LOS. Although advanced age was associated with prolonged hospitalization in univariate analysis, in multiple regression the significant predictive value was not reached.

A frailty marker, Charlson comorbidity index was not associated with extended LOS in our analyses. In a study of 302 AF patients extended LOS was associated with frailty and advanced age, however, the cohort was overall much older compared to our study sample (84.7 ± 7.1 compared to 72.5 ± 10.3 years) [33].

### 4.2. Severity of Atrial Fibrillation Burden

Regarding the duration of hospitalization and the type of AF, there was no independent association in our study, echoing the results reported by Steinberg et al., showing no correlation between the type of AF and LOS [8].

Increased heart rate was significantly correlated with hospital stay in AF patients with or without concomitant HF, in an analysis by Steinberg et al. [8]. However, in our study, baseline heart rate was poorly correlated with prolonged LOS, irrespective of the AF type and was outperformed by other stronger predictors in multivariable analysis.

Type of anticoagulant medication was not linked to extended LOS, unlike previous reports associating NOACs with shorter hospitalizations as opposed to warfarin [34,35,36]. We hypothesize this is probably due to their increasing prescription in our cohort compared to preceding registry reports [37,38]. In addition, part of the ABC-pathway [39], the use of rate or rhythm directed medical therapy or emergency cardioversion did not influence LOS, while elective electrical cardioversion was linked to shorter hospitalization.

### 4.3. Cardiac Substrate Characteristics

HF is an independent predictor of prolonged LOS in hospitalized cardiac patients [40]. In a long-term registry of more than 900,000 patients, Ziff et al., showed that patients with AF and HF were at greater risk for longer hospital stay compared to either condition alone [41]. Similar results were also reported from the ORBIT-AF registry, where significant HF at baseline (NYHA Class II or higher) had a major impact on LOS [8]. The bidirectional relation between AF and HF was an important determinant of prolonged hospitalization in our study, with HF affecting more than 80% of patients. The highest independent risk of extended LOS was associated to ADHF as well as HF severity markers, respectively, elevated NT-proBNP levels and reduced EF.

NT-proBNP levels, as a surrogate for intracardiac volumes and filling pressures, with clinical utility not only in early diagnosis but also in risk stratification in HF [42], represented an independent predictor of extended LOS in our study. Previous data also classified higher NT-proBNP values as predictors of prolonged hospitalization in a HF cohort including more than 70,000 patients [25]. NT-proBNP guided HF therapy could, therefore, be a successful intervention in preventing re-admissions, as well as titrating in-hospital therapy to ensure adequate decongestion [42].

Patients with AF and ACS had the highest risk of prolonged hospitalization, after adjustment for all other identified risk factors in multivariate analysis. The mutual relation was also highlighted in a recent analysis of the AMIS Plus registry [43]. Out of more than 35,000 patients presenting with ACS, extended LOS was observed in those with pre-existing and new-onset AF compared to those with sinus rhythm [43]. Moreover, preexisting AF was an independent predictor of in hospital mortality in this study [43]. Earlier reports also stated that ACS patients with either new-onset or previously diagnosed AF were older and had higher ACS related risk [44]. Therefore, this aggregate disease burden increases the risk of extended LOS by incrementing case complexity and potential complications.

An analysis from the ROCKET-AF registry showed that infections were an important cause (47%) of AF hospitalization [45]. Results from the EORP on Heart Failure—Polish Registry, a cohort of 1126 HF patients, likewise, confirmed the influence of infections on prolonged hospitalization [21]. Similarly, our research identified concomitant infections as independent predictors of extended LOS.

Defining the patient profile associated with extended hospitalization in AF allows not only a better understanding of disease kinetics, but also the recognition of case severity patterns, as well as the improvement of alternative strategies of management. Identifying the modifiable factors associated with prolonged LOS and consequently their correction may improve the hospitalization period. As previously determined, extended LOS can be regarded as a marker of poor prognosis in cardiovascular diseases, associated with doubled mortality during a mean follow-up period of 414 ± 121 days [21]. In our study of AF patients, concurring with previous reports predominantly of AF patients and other cardiac diseases, prolonged hospitalization was due to the association of cardiovascular comorbidities, respectively ACS and ADHF, as well as infections, combined with a higher HAS-BLED score. We highlighted the importance of HF clinics as well as upgraded and specialized cardiovascular outpatient care, as previously suggested [40], since prevention of complications and progressing severity of cardiac conditions is of utmost importance for the overall prognosis of these patients.

### 4.4. Limitations

There are several limitations to our research. It is a retrospective observational study, conducted in a single center. However, we consider it is representative for a larger population, given the wide addressability of patients from adjacent areas as well other regions in the country.

Data about previous or consequent hospitalizations was scarce, therefore, their impact in predicting extended LOS could not be assessed properly. Accordingly, these variables were not included in the analysis.

Patients with stroke or bleeding complications were not included in the study, since they were not hospitalized in the cardiology department.

## 5. Conclusions

In our study, in AF inpatients, the main drivers of hospitalization were cardiovascular disease burden, comorbidities and infections, and not the AF specific clinical or therapeutical characteristics. The independent predictors of extended LOS were ACS, ADHF, HFrEF, increased NT-proBNP levels, infections, and elevated HAS-BLED score. AF patients with extended LOS had a cluster of illnesses, were overall sicker and showed more signs of cardiac disease severity. Interventions that could optimize length of hospitalization include improved prevention strategies for IHD, optimized HF outpatient care, periodical assessment and correction of modifiable HAS-BLED parameters and prevention and early detection of infections.

## Figures and Tables

**Table 1:** Baseline characteristics of patients stratified by LOS.

	Total *n* = 949	LOS < 7 Days *n* = 659	LOS ≥ 7 Days *n* = 290	*p*-Value
**Demographics**				
Age, years, mean ± SD	72.5 ± 10.4	71.87 ± 10.5	73.9 ± 9.6	0.005
Female gender, *n* (%)	509 (52.9%)	350 (53.1%)	152 (52.4%)	0.69
**AF characteristics**				
Type of AF				
Paroxysmal, *n* (%)	295 (31.1%)	210 (31.9%)	85 (29.3%)	0.433
Persistent, *n* (%)	229 (24.1%)	162 (24.6%)	67 (23.1%)	0.249
Permanent, *n* (%)	425 (44.8%)	287 (43.6%)	138 (47.6%)	0.624
AF on admission	724 (76.3%)	490 (74.4%)	234 (80.7%)	0.04
Risk scores				
CHA_2_DS_2_ VASc, median [IQR]	4 [3, 5]	4 [3, 5]	5 [4, 6]	<0.001
HAS BLED, median [IQR]	1 [1, 1]	1 [1, 1]	1 [1, 2]	<0.001
Emergency AF Cardioversion	60 (6.3%)	39 (5.9%)	21 (7.2%)	0.53
**HF characteristics**				
HF, *n* (%)	786 (82.9%)	528 (80.2%)	258 (89.0%)	<0.001
ADHF, *n* (%)	383 (40.4%)	210 (31.9%)	173 (59.9%)	<0.001
Type of HF				
HFpEF, *n* (%)	456 (55.1%)	341 (61.2%)	115 (42.6%)	<0.001
HFmrEF, *n* (%)	157 (19.0%)	104 (18.7%)	53 (19.6%)	0.742
HFrEF, *n* (%)	214 (25.9%)	112 (20.1%)	102 (37.8%)	<0.001
NYHA class				
I-II, *n* (%)	517 (54.5%)	395 (59.9%)	122 (42.1%)	<0.001
III-IV, *n* (%)	267 (28.1%)	132 (20.0%)	135 (46.6%)	<0.001
**Comorbidities and cardiovascular risk factors**				
Charlson comorbidity index	4 [3, 5]	4 [3, 5]	4 [3, 4]	0.178
IHD, *n* (%)	307 (32.4%)	192 (29.2%)	115 (39.7%)	0.001
Prior MI, *n* (%)	99 (10.4%)	56 (8.5%)	43 (14.8%)	0.003
ACS, *n* (%)	26 (2.7%)	11 (1.7%)	15 (5.2%)	0.002
Hypertension, *n* (%)	764 (80.6%)	551 (83.7%)	213 (73.4%)	<0.001
Dyslipidemia, *n* (%)	637 (68.0%)	444 (68.2%)	193 (67.5%)	0.828
Diabetes Mellitus, *n* (%)	297 (31.3%)	190 (28.8%)	107 (36.9%)	0.014
Anemia, *n* (%)	275 (29.4%)	168 (25.9%)	107 (37.3%)	<0.001
History of Stroke/ TIA, *n* (%)	137 (14.5%)	82 (12.5%)	55 (19.0%)	0.009
Obesity, *n* (%)	295 (31.1%)	212 (32.2%)	83 (28.6%)	0.27
CKD, *n* (%)	341 (36.0%)	222 (33.7%)	191 (41.0%)	0.031
Dementia, *n* (%)	35 (3.7%)	17 (2.6%)	18 (6.2%)	0.006
**Clinical data**				
Chief complaints				
Dyspnea	421 (44.3%)	243 (36.9%)	178 (61.4%)	<0.001
Chest pain	36 (3.8%)	19 (2.9%)	17 (5.9%)	0.04
Hypertensive emergency	76 (8.0%)	64 (9.7%)	12 (4.1%)	0.005
Sincope/presincope	49 (5.2%)	36 (5.5%)	13 (4.5%)	0.64
Fatigue	138 (14.5)	105 (15.9%)	33 (11.4%)	0.08
Palpitations	85 (8.9%)	69 (10.5%)	16 (5.5%)	0.02
HR, bpm, mean ± SD	85.03 ± 27.33	83.01 ± 25.73	89.55 ± 30.17	<0.001
**Biological data**				
NT-proBNP, pg/mL, median [IQR]	1771 [793.6, 3520]	1395 [629.7, 2700]	2905 [1429, 5597]	<0.001
eGFR, mL/min/1.73 m^2^, median [IQR]	70.14 [52.27, 87.18]	71.46 [54.05, 88.96]	66.95 [46.04, 82.76]	<0.001
INR *, median [IQR]	1.51 [1.16, 2.28]	1.53 [1.15, 2.25]	1.47 [1.20, 2.42]	0.27
Hb, g/dL, median [IQR]	13.4 [12.0, 14.5]	13.5 [12.3, 14.6]	13.1 [11.7, 14.3]	0.001
**Echocardiographic parameters**				
LA, mm, mean ± SD	46.9 ± 7.86	46.1 ± 7.5	48.4 ± 8.3	<0.001
EF, %, mean ± SD	45.8 ± 13.4	48.0 ± 12.1	41.4 ± 14.8	<0.001
**Medication**				
Anticoagulant therapy				
NOACs, *n* (%)	479 (51.5%)	345 (53.5%)	134 (47.0%)	0.07
VKA, *n* (%)	427 (45.9%)	285 (44.2%)	142 (49.8%)	0.12
All other cardiovascular medication				
Antiplatelet therapy, *n* (%)	61 (6.4%)	45 (6.8%)	16 (5.6%)	0.47
Beta-blockers, *n* (%)	705 (75.5%)	489 (75.3%)	216 (75.8%)	0.85
ACEI or ARB, *n* (%)	756 (81.0%)	531 (81.9%)	225 (78.9%)	0.28
Diuretics, *n* (%)	706 (76.2%)	483 (75.2%)	223 (78.5%)	0.28
MRA, *n* (%)	273 (29.9%)	161 (25.6%)	112 (39.4%)	<0.001
Digoxin, *n* (%)	62 (6.6%)	41 (6.3%)	21 (7.4%)	0.56
Statins, *n* (%)	557 (59.6%)	389 (59.9%)	168 (58.9%)	0.776
Antiarrhythmic drugs, *n* (%)	103 (11.4%)	73 (11.7%)	30 (10.6%)	0.07

ACEI, angiotensin converting enzyme inhibitors; ACS, acute coronary syndrome; ADHF, acute decompensated heart failure; AF, atrial fibrillation; ARB—angiotensin receptor blocker; CKD, chronic kidney disease; eGFR, estimated glomerular filtration rate; EF, ejection fraction; HF, heart failure; HFmrEF, heart failure with mid-range ejection fraction; HFpEF, heart failure with preserved ejection fraction; HFrEF, heart failure with reduced ejection fraction; HR, heart rate; IHD, ischemic heart disease; IQR, interquartile range; LA, left atrium; LOS, length of hospital stay; MI, myocardial infarction; MRA—mineralocorticoid receptor antagonist; NOACs, non-vitamin K antagonist oral anticoagulants; NYHA, New York Heart Association; TIA, transient ischemic attack; VKA, vitamin K antagonist. * Analysis of 479 patients receiving VKA therapy.

**Table 2:** Determinants of prolonged LOS—univariate analysis.

	RR (95% CI)	*p*-Value
Age	1.10 (1.01–1.20)	0.02
AF on admission	1.11 (1.01–1.21)	0.04
Palpitations on admission	0.84 (0.75–0.94)	0.02
HF	1.19 (1.09–1.30)	<0.001
ADHF	1.44 (1.31–1.60)	<0.001
Dyspnea at rest	2.82 (1.41–5.65)	<0.001
HFrEF	1.38 (1.20–1.59)	<0.001
IHD	1.16 (1.05–1.28)	<0.001
Prior MI	1.25 (1.04–1.49)	0.003
ACS	1.65 (1.05–2.60)	0.002
Chest pain on admission	1.33 (0.97–1.81)	0.04
Hypertensive emergency	0.81 (0.73–0.90)	0.005
Diabetes mellitus	1.12 (1.01–1.24)	0.01
TIA/stroke	1.18 (1.02–1.37)	0.008
CKD < 60 mL/min/1.73 m^2^	1.10 (1.01–1.20)	0.03
Anemia	1.19 (1.07–1.32)	<0.001
Dementia	1.44 (1.02–2.03)	0.006
Infection	1.48 (1.26–1.73)	<0.001

Abbreviations: ACS, acute coronary syndrome; ADHF, acute decompensated heart failure; CI, confidence interval; CKD—chronic kidney disease; HF, heart failure; HFrEF, heart failure with reduced ejection fraction; IHD, ischemic heart disease; MI, myocardial infarction; RR, risk ratio; TIA, transient ischemic attack.

**Table 3:** Determinants of prolonged LOS-ROC curve analysis.

	AUC (95% CI)	Cut-Off Value	*p*-Value
Heart rate	0.56 (0.53–0.59)	>104 bpm	0.005
NT-proBNP	0.69 (0.66–0.73)	>1986 pg/mL	<0.001
EF	0.63 (0.6–0.67)	<44%	<0.001
CHA2DS2-VASC	0.58 (0.55–0.61)	>4	<0.001
HAS-BLED	0.58 (0.54–0.61)	>3	<0.001

Abbreviations: AUC, area under the curve; CI, confidence interval; EF, ejection fraction.

**Table 4:** Determinants of prolonged LOS—multivariable analysis.

	HR	95% CI	*p*-Value
ACS ^a^	4.60	1.66–12.69	0.003
Infections ^a^	2.61	1.44–3.23	<0.001
NT-proBNP > 2008 ng/mL ^a^	1.96	1.37–2.82	<0.001
ADHF ^a^	1.76	1.23–2.51	0.002
HFrEF ^a^	1.69	1.15–2.47	0.007
HAS-BLED score ^b^	1.42	1.14–1.78	<0.001

^a^ Dichotomous variable; ^b^ continuous variable—HR calculated for each point increase in score value; Abbreviations: ACS, acute coronary syndromes; ADHF, acute decompensated heart failure; CI, confidence interval; HFrEF, heart failure with reduced ejection fraction; HR, hazard ratio. Variables included in the regression without independent significance (alphabetical order): age, anemia, CHA_2_DS_2_-VASc score, chest pain on admission, dementia, diabetes mellitus, dyspnea at rest, heart failure, heart rate on admission, history of myocardial infarction, history of stroke/ ischemic transient attack, hypertensive emergency, ischemic heart disease, left atrial diameter, palpitations on admission, permanent atrial fibrillation, reduced glomerular filtration rate < 60 mL/min, stable angina.

## Data Availability

Data used in this study may be provided by the authors upon reasonable request.

## Supplementary Materials

The following are available online at https://www.mdpi.com/article/10.3390/jcm10163715/s1, Table S1: baseline characteristics of patients stratified by AF type, Table S2: risk score components and prolonged hospitalization.
