# Supplementary material for: Determinants of Prolonged Length of Hospital Stay of Patients with Atrial Fibrillation
Source: J Clin Med. 2021 Aug 20;10(16):3715. doi: 10.3390/jcm10163715 (PMC8396858; doi:10.3390/jcm10163715)
Supplement: Supplementary file 1 [file jcm-10-03715-s001.zip › jcm-1305495-supplementary.pdf]

Supplementary Materials

Table S1. Baseline characteristics of patients stratified by AF type.

	**Paroxysmal AF** *n* = 295	**Persistent AF** *n* = 229	**Permanent AF** *n* = 425	***p*-Value**
**Demographics**				
Age, years	70.5 ± 10.6	69.7 ± 10.8	75.3 ± 9.14	<0.001
Female Gender, *n* (%)	170 (57.6%)	107 (46.7%)	225 (52.9%)	0.04
LOS, days, median [IQR]	4 [2, 7]	4 [3, 7]	5 [3, 7]	0.01
**HF Characteristics**				
HF, *n* (%)	192 (65.1%)	194 (84.7%)	400 (94.3%)	<0.001
ADHF, *n* (%)	60 (20.5%)	97 (42.4%)	226 (53.2%)	<0.001
Type of HF				
HFpEF, *n* (%)	168 (70.9%)	107 (52.7%)	181 (46.8.0%)	<0.001
HFmrEF, *n* (%)	33 (13.9%)	28 (13.8%)	96 (24.8%)	<0.001
HFrEF, *n* (%)	36 (15.2%)	68 (33.5%)	110 (28.4%)	<0.001
NYHA class				
I-II, *n* (%)	142 (48.1%)	122 (53.3%)	253 (59.5%)	0.01
III-IV, *n* (%)	49 (16.6%)	70 (30.6%)	148 (34.8%)	0.01
**AF cardioversion**				
Emergency cardioversion, *n* (%)	12 (4.1%)	39 (17.0%)	9(2.1%)	<0.001
**Comorbidities and risk factors**				
Ischemic Heart Disease, *n* (%)	88 (29.9%)	63 (27.5%)	156 (36.7%)	0.03
Prior MI, *n* (%)	33 (11.2%)	23 (10.0%)	43 (10.1%)	0.88
ACS, *n* (%)	14 (4.6%)	5 (2.2%)	7 (1.6%)	0.04
Hypertension, *n* (%)	246 (83.7%)	182 (79.5%)	336 (79.1%)	0.27
Dyslipidemia, *n* (%)	212 (73.1%)	154 (67.8%)	271 (64.5%)	0.03
Diabetes Mellitus, *n* (%)	84 (28.5%)	71 (31.4%)	142 (33.4%)	0.37
Anemia, *n* (%)	77 (26.6%)	54 (23.9%)	144 (34.3%)	0.01
History of Stroke/ TIA, *n* (%)	36 (12.2%)	35 (15.3%)	66 (15.5%)	0.43
Obesity, *n* (%)	83 (28.2%)	80 (34.9%)	132 (31.1%)	0.26
Chronic Kidney Disease, *n* (%)	96 (32.5%)	70 (30.6%)	175 (41.3%)	0.008
Dementia, *n* (%)	13 (4.4%)	3 (1.3%)	19 (4.5%)	0.02
**Current medication**				
Anticoagulant therapy				
NOACs, *n* (%)	176 (61.1%)	140 (62.5%)	163 (39.0%)	<0.001
VKA, *n* (%)	102 (35.4%)	79 (35.3.%)	246 (58.9%)	<0.001
All other cardiovascular medication				
Antiplatelet therapy, *n* (%)	24 (8.2%)	18 (7.9%)	19 (4.5%)	0.07
Beta-Blockers, *n* (%)	209 (72.1%)	177 (78.7%)	319 (76.1%)	0.20
ACEI or ARB, *n* (%)	231 (79.7%)	179 (79.9%)	346 (82.6%)	0.55
Diuretics, *n* (%)	168 (58.9%)	172 (77.1%)	366 (87.6%)	<0.001
MRA, *n* (%)	43 (15.3%)	79 (36.2%)	151 (36.6%)	<0.001
Digoxin, *n* (%)	6 (2.1%)	10 (4.4%)	46 (11.0%)	<0.001
Statins, *n* (%)	185 (63.8%)	137 (60.9%)	235 (56.1%)	0.11
Antiarrhythmic drugs, *n* (%)	40 (14.4%)	34 (15.6.%)	29 (7.1%)	0.001
**Clinical data**				
Heart Rate, bpm, median [IQR]	72 [61, 95]	89 [70, 118]	80 [70, 100]	<0.001

**Biological data**				
NT-proBNP, pg/ml, median [IQR]	834 [317.3, 2229]	2142 [1112, 4057]	2092 [1102, 3893]	<0.001
eGFR, ml/min/1.73 m^2, median [IQR]	74.8 [53.6, 90.7]	73.0 [54.5, 88.0]	66.3 [50.5, 84.1]	0.005
INR [a], median [IQR]	1.26 [1.06, 1.78]	1.30 [1.11, 1.90]	1.87 [1.36, 2.61]	<0.001
Hb, g/dl, median [IQR]	13.4 [12.2, 14.4]	13.6 [12.4, 14.9]	13.1 [11.8, 14.3]	<0.001
**Echocardiographic parameters**				
LA, mm, mean [SD]	43.1 ± 7.0	45.8 ± 7.1	49.6 ±.7.7	<0.001
EF, %, mean [SD]	50.1 ± 12.1	44.8 ±14.6	44.1 ± 13.07	<0.001

[a] Values obtained from 454 patients receiving VKA; Abbreviations: ACEI, angiotensin converting enzyme inhibitors; ACS, acute coronary syndrome; ADHF, acute decompensated heart failure; AF, atrial fibrillation; ARB, angiotensin receptor blocker; eGFR, estimated glomerular filtration rate; EF, ejection fraction; HF, heart failure; HFmrEF, heart failure with mid-range ejection fraction; HFpEF, heart failure with preserved ejection fraction; HFrEF, heart failure with reduced ejection fraction; LA, left atrium; LOS, length of hospital stay; MI, myocardial infarction; MRA, mineralocorticoid receptor antagonist; NOACs, non vitamin K antagonist oral anticoagulants; NYHA, New York Heart Association; TIA, transient ischemic attack; VKA, vitamin K antagonist.

Table S2. Risk score components and prolonged hospitalization.

**CHA_2DS_2-VASc**				
	**Prevalence *n* (%)**	**Risk ratio**	**95%CI**	***p*-Value**
Congestive heart failure	786 (82.9%)	1.20	1.09–1.30	<0.001
Hypertension	764 (80.6%)	0.80	0.70–0.91	<0.001
Age ≥ 75 years old	434 (45.7%)	1.10	1.01–1.20	0.02
Diabetes	297 (31.3%)	1.12	1.01–1.24	0.01
Stroke	137 (14.5%)	1.18	1.02–1.37	0.008
Vascular disease	328 (34.6%)	1.16	1.06–1.28	<0.001
Age 65–74 years old	787 (75.9%)	1.12	1.04–1.22	0.01
Sex category (female)	447 (47.1%)	1.00	0.92–1.09	0.84
**HAS-BLED**				
	**Prevalence *n* (%)**	**Risk ratio**	**95%CI**	***p* value**
Hypertension, uncontrolled	97 (10.7%)	1.00	0.87–1.15	0.94
Abnormal kidney function	20 (2.1%)	1.40	0.90–2.16	0.06
Abnormal liver function	25 (2.6%)	1.34	0.92–1.96	0.05
Stroke	137 (14.5%)	1.18	1.02–1.37	0.01
Bleeding tendency	9 (0.95%)	0.89	0.62–1.26	0.58
Labile INR [a]	61 (8.1%)	1.16	0.93–1.44	0.12
Elderly (age > 65 years old)	758 (73.2%)	1.13	1.04–1.22	0.01
Drugs predisposing to bleeding [b]	61 (6.5%)	0.94	0.80–1.09	0.47

[a] Evaluated in patients receiving vitamin K antagonists (*n* = 811); [b] Evaluated in patients receiving oral anticoagulant therapy (*n* = 1245).
